# Evidence for a Role of Oxidative Stress in the Carcinogenicity of Ochratoxin A

**DOI:** 10.1155/2011/645361

**Published:** 2011-06-22

**Authors:** M. Marin-Kuan, V. Ehrlich, T. Delatour, C. Cavin, B. Schilter

**Affiliations:** Chemical Food Safety Group, Quality & Safety Department, Nestlé Research Center, P.O. Box 44, Vers-chez-les-Blanc, 1000 Lausanne 26, Switzerland

## Abstract

The *in vitro* and *in vivo* evidence compatible with a role for oxidative stress in OTA carcinogenicity has been collected and described. Several potential oxido-reduction mechanisms have been identified in the past. More recently, the possibility of a reduction of cellular antioxidant defense has been raised as an indirect source of oxidative stress. Consequences resulting from the production of oxidative stress are observed at different levels. First, OTA exposure has been associated with increased levels of oxidative DNA, lipid, and protein damage. Second, various biological processes known to be mobilized under oxidative stress were shown to be altered by OTA. These effects have been observed in both *in vitro* and *in vivo* test systems. *In vivo*, active doses were often within doses documented to induce renal tumors in rats. In conclusion, the evidence for the induction of an oxidative stress response resulting from OTA exposure can be considered strong. Because the contribution of the oxidative stress response in the development of cancers is well established, a role in OTA carcinogenicity is plausible. Altogether, the data reviewed above support the application of a threshold-based approach to establish safe level of dietary human exposure to OTA.

## 1. Introduction

Ochratoxin A (OTA) is a mycotoxin produced by several food-borne species of *Aspergillus *and *Penicillium* fungi. OTA occurs in various food materials and therefore humans are continuously exposed to relatively small amounts of it. Because of its wide occurrence and consequent exposure, together with a potent carcinogenic potential in animal models, OTA has attracted significant public health attention over the last few years. Several national and international food safety organizations and expert groups have conducted a thorough review of the situation as well as risk assessments in order to provide an insight on the health significance of OTA in food. 

Human epidemiology has been inconclusive: a number of studies have suggested a correlation between exposure to OTA and Balkan Endemic Nephropathy (BEN) and mortality from urinary tract tumors [1, 2]. Epidemiological data were recently reviewed by several expert groups [3–6]. All concluded that causality between intake of OTA and human nephropathy could not be established. Therefore, the IARC statement that there is inadequate evidence for carcinogenicity in humans (group 2B) [7] appears still valid. Recently, other nephrotoxic agents have been put forward as the primary cause of BEN [6, 8, 9].

In absence of adequate human data, risk assessments have relied on animal data. Kidney has been considered as the key target organ of OTA toxicity. In all animal species studied, OTA was found to produce renal toxicity, while in rodents renal carcinogenicity was clearly established. Recently, OTA renal and hepatic carcinogenicity was also observed in chicks [10]. Using a LOAEL of 8 mcg/kg bw/day based on early markers of renal toxicity in pig (the most sensitive animal species) and applying an uncertainty factor of 450, EFSA [4] allocated a Tolerable Weekly Intake (TWI) of 120 ng/kg bw. Analysis of dietary exposure throughout Europe revealed that the current average OTA exposure (50–60 ng/kg bw/week) is well within the TWI [4]. The joint FAO/WHO Expert Committee on Food Additives (JECFA) first evaluated OTA at their 37th meeting [11]. Based on the LOAEL in pig, and applying an uncertainty factor of 500, JECFA allocated a Provisional Tolerable Weekly Intake (PTWI) of 112 ng/kg bw [11]. This value was rounded to 100 ng/kg bw/week and confirmed in several subsequent meetings [12, 13].

In 2008, JECFA applied a benchmark dose (BMD) modeling approach using carcinogenicity data [14]. The BMD is the dose estimated to cause a predefined increase (e.g., 10% for the BMD10) in tumor incidence over background. The BMDL is the lower limit of a one-sided 95% confidence interval of the BMD. The occurrence of combined adenomas and carcinomas in the kidneys of male rats was considered by JECFA to be the most appropriate data for modeling. Values of 18–33 mcg/kg bw/d and 15–25 mcg/kg bw/day were calculated for, respectively, the BMD10 and BMDL10. Because the BMD approach did not provide a lower point of departure than the LOAEL in pig, JECFA decided to reconfirm the PTWI of 100 ng/kg bw/day [14]. 

A recent health risk assessment, performed by Health Canada [15] recommends to regulate OTA as a nonthreshold carcinogen, because of the uncertainties regarding the mode of action. The authors defined a negligible cancer risk intake (NCRI, risk level 1 : 100 000) using the tumorigenic dose at which 5% of rats are likely to develop tumors (TD_05_, derived through modeling) as point of departure. Importantly, there is considerable convergence between the NCRI established in this assessment and the TDI derived by EFSA.

In the risk assessment of carcinogenic substances, consideration of the mode of action (MOA) is essential, determining the method to be applied in order to define levels of exposure below which a low safety concern is expected. The key events analysis framework of the MOA has not yet been formally applied to OTA. However, the approach used by most expert groups (EFSA, JECFA, ILSI) to establish the safe level of exposure of OTA (based on uncertainty factors) implies the consideration of key events compatible with a threshold effect. For these groups, amongst the mechanisms of action highlighted as possible, oxidative stress has been presented as one of the most probable [5, 14]. 

### 1.1. Scope of the Paper

Over the last decades, studies aimed at elucidating the modes of action implicated in OTA toxicity and carcinogenicity have been published [16]. There has been considerable debate for many years over the genotoxicity of OTA and its actual role in carcinogenicity [3–5, 11–15, 17, 18]. 

Although genotoxicity is likely to play a role in OTA carcinogenicity [3, 4], the actual molecular mechanism involved, either through covalent adduct formation, through other indirect modifications, or both is still unclear. The potential of OTA to form covalent DNA adducts has been subjected to debate due to conflicting data in the literature. Using ^32^P-postlabelling analysis, large numbers of putative OTA-derived DNA adducts have been reported to be present in a wide range of tissues from OTA-treated rats, mice as well as pigs [2, 17, 19–22]. However, so far, these adducts have never been observed by any other highly specific techniques such as radioactivity measurements using ^3^H-labelled OTA (^3^H-OTA) [23], accelerator mass spectrometry (AMS) [24], or liquid chromatography-tandem mass spectrometry (LC-MS/MS) [25]. 

The present paper is not intended to provide a thorough review of the complex and controversial scientific literature on DNA-adduct formation. However, it is important to keep in mind that DNA adducts are increasingly considered as markers of exposures and not only of effects [26, 27] and that DNA-covalent binding does not necessarily determine the shape of the dose-response at low level of exposure [28, 29]. According to Mantle and coworkers [21, 30], experimental dose-response data for OTA's renal carcinogenesis makes a compelling case for OTA being a thresholded carcinogen in male rat. In this context, it appears important to also consider other potential modes of action, which could potentially contribute to OTA carcinogenicity. In the near future, the application of the mode of action framework [28] will likely help to understand the individual contribution of all mechanisms described up to now for OTA.

The focus of the present short paper was to collect and highlight the evidence associated with a role for oxidative stress as a plausible mechanism to consider for OTA. A list of the studies used to illustrate the main messages of the present paper is provided in Table 1. Although not exhaustive, the list shows that over the last two decades, numerous investigators have documented the generation of oxidative stress as a result of OTA treatment in both *in vitro* and *in vivo* model systems.

## 2. Sources of OTA-Mediated Oxygen-Species Generation

Production of reactive oxygen species (ROS) leading to oxidative stress and macromolecular damage is known to contribute to the pathogenesis of age-related as well as chronic diseases including cancer [31–35]. A number of studies are available documenting that OTA is associated with the production of reactive oxygen species and resulting oxidative stress through various direct and indirect mechanisms. 

### 2.1. Oxido-Reduction Mechanisms

Several oxido-reduction mechanisms elicited by OTA have been proposed. In a reconstituted system consisting of phospholipid vesicles, the flavoprotein NADPH-cytochrome P450 reductase and Fe^3+^, OTA was found to chelate ferric ions (Fe^3+^), facilitating their reduction to ferrous ions (Fe^2+^), which in the presence of oxygen, provided the active species initiating lipid peroxidation [36]. Results indicated that the hydroxyl radical was not involved in the process. A role for cytochrome P450 in this reaction was also suggested [36]. In contrast, others found that OTA induced oxidative damage through the generation of hydroxyl radicals. This reaction conducted with microsomes, in presence of NADPH and O_2_ did not require exogenous Fe [20]. Structure-activity studies have also suggested that the toxicity of OTA may be attributable to its isocoumarin moiety and that the lactone carbonyl group may be involved in its toxicity. Using a *Bacillus brevis* model, Hoehler's et al. showed that OTA behaved as a cell pro-oxidant through mobilization of Fe^2+^ and Ca^2+^ pathway leading to uncoupling oxidative phosphorylation and increased production of hydroxyl radical via the Fenton reaction [37]. However, in other studies using OTA and structural analogs, a direct correlation between toxicity and iron chelating capacity was only partially supported [38].

The generation of an OTA hydroquinone/quinone couple from the oxidation of OTA (phenol oxidation) by electrochemical, photochemical, and chemical processes was reported [39, 40]. The quinone is thought to undergo reductions to form hydroquinone, postulated to be responsible for the formation of the glutathione conjugate of OTA. Such events are likely to result in redox cycling and in the generation of reactive oxygen species [20, 40, 41]. The formation of OTA-derived quinones has been observed in cell cultures *in vitro* [41] as well as *in vivo* [42, 43].

### 2.2. Reduction of Antioxidant Defenses

OTA was found to reduce the expression of several genes regulated by nuclear factor-erythroid 2 p45-relatetd factor (Nrf2) [44–47]. This was observed at the RNA and protein levels, both *in vitro* and *in vivo* test systems. Nrf2 is involved in both the basal expression as well as in the induction of genes encoding detoxification, cytoprotective, and antioxidant enzymes [48, 49]. A reduction of the expression of these genes is likely to result in decrease in antioxidant defenses leading to oxidative stress and macromolecular damage. This was confirmed with OTA. Through *in vitro* and *in vivo* studies, a correlation was observed between the OTA-dependent reduction of the Nrf2 pathway and an increased production of oxidative damage [46]. In this context, it is interesting to note that OTA was found to increase the expression of inducible nitric oxide synthase (iNOs) [50–52], an enzyme responsible for the production of nitric oxide (NO). An association between iNOs expression and the development of cancers was suggested in humans and animals *in vivo* [53]. In excess, NO may behave as a toxic radical producing nitrosative stress. NO is known to react with oxygen anion radical superoxide to form the pro-oxidant peroxynitrite. Under physiological conditions, peroxynitrite rapidly decomposes to generate a nitro radical intermediate leading to protein and DNA nitration. OTA was shown to stimulate protein and possibly DNA nitration [52, 53] indicating that OTA exposure may be considered as a source of both oxygen and also reactive nitrogen radicals/species (RNS).

## 3. OTA-Mediated Oxidative Damage

### 3.1. DNA-Damage

ROS, such as hydroxyl radicals and nitric oxide, are capable of forming oxidized DNA bases that directly produce diverse types of DNA damage [54–56]. The oxidized DNA bases appear to be capable of inducing mutations that are commonly observed in neoplasia [33]. As illustrated in Table 1, diverse biomarkers have been analyzed showing that OTA induces DNA damage. In cell cultures, the OTA-dependent production of ROS was correlated with an increased formation of 8-oxoguanine [57–59]. Moreover, OTA was shown to induce DNA strand breaks as assessed by comet assay in liver, kidney, and spleen of F344 rats given 0, 0.25, 0.50, 1.0, and 2.0 mg/kg bw/day by gavage for 2 weeks [60]. In liver and kidney, the extent of DNA damage analyzed by comet assay was further enhanced in the presence of formamidopyrimidine glycosylase (Fpg), an enzyme involved in excision repair of oxidized DNA bases [57, 58, 61]. Another study [62] detected DNA-strand breaks (using the comet assay) in the kidney of female Wistar rats treated intraperitoneally with OTA (0.5 mg/kg bw/day for 7, 14, and 21 days; *n* = 5 per group). The severity of the DNA lesions in the kidney increased according to the OTA dose and was at maximum after 21 days of treatment. Other authors have observed oxidative DNA-damage in various tissues of animals treated with a wide range of OTA doses and treatment durations [46, 60, 63, 64].

### 3.2. Protein Damage

Carbonylation of proteins occurs through a variety of oxidative pathways [54, 65–67]. Carbonylation is an important protein modification associated with alterations of protein (enzymes) function, protein misfolding, protein fate, and proteolysis. An increase in protein carbonyl content of tissues has been associated with a number of pathological disorders. Due to their abundance in mammalian cells, cytoskeletal proteins like actin are common targets for a variety of ROS and low-molecular weight reactive carbonyl species [67]. Inconsistent data have been reported on the potential impact of OTA on protein carbonylation [52, 59, 63, 68, 69]. No increase in protein oxidation was observed in liver and kidney of F344 rats treated with OTA at 0.3 mg/kg bw per day for 4 weeks [69]. The same finding was reported in liver of Wistar rats treated with OTA at 289 *μ*g/kg bw for 90 days [63]. In contrast, in another *in vivo* study measuring protein oxidation in Wistar rats treated with OTA (0.5 mg/kg bw/day) for 24 h, 7, 14 and 21 days, a significant increase in protein carbonyls was found after 14 and 21 days of treatment in, respectively, the kidney and the liver [68]. Oxidative protein modification was observed in vitro [52].

### 3.3. Lipid Damage

Lipid peroxidation is among the most extensively investigated processes induced by free radicals. Of these, the by-products, 4-hydroxy-2-nonenal (HNE), the tautomer malondialdehyde (MDA), acrolein and crotonaldehyde have been widely studied. The ability of these reactive electrophiles to modify DNA bases, yielding promutagenic lesions, is considered to contribute to the mutagenic and carcinogenic effects associated with oxidative stress-induced lipid peroxidation. HNE and MDA have increasingly been implicated in carcinogenesis [33, 70–72]. OTA has been reported to increase MDA formation. Initially, Rahimtula's group [73] observed that OTA was able to stimulate lipid peroxidation when added to liver or kidney microsomes or when administered to rats *in vivo*. Stimulation of lipid peroxidation by OTA-iron complex facilitating the reduction of iron was further reported [36]. Moreover, additional studies [74] indicated that OTA induced lipid peroxidation accompanied by leakage of calcium from calcium-loaded microsomes. Increased formation of MDA was observed in animal models treated orally with different doses of OTA [75–79]. HNE-protein adducts were measured in cell cultures treated with OTA [52].

## 4. OTA-Mediated Biological Response

It is widely acknowledged that reactive oxygen and nitrosative species can trigger biological responses such as stimulation or inhibition of signal transduction and gene expression. Such biological responses are considered to contribute to the expression of the carcinogenic potential of the reactive chemicals. A number of *in vitro* and *in vivo* studies are consistent with a role of oxidative and probably nitrosative stress as messengers involved in the adverse biological effects of OTA (Table 1).

## 5. Cell Signaling

ROS induces release of calcium from intracellular stores, resulting in the activation of kinases, such as protein kinase C (PKC). ROS species play also a critical role in the selective mobilization of other cell signaling responses. Cell signaling phosphoproteins of mitogen-activated protein (MAP) kinases including ERK, c-Jun N-terminal kinases (JNK), and p38 kinases are involved in proliferation, differentiation, and apoptosis. Activation of these molecules has been observed in response to changes in the cellular redox balance and are considered as vectors of ROS biological effects [33]. *In vitro* experiments provided evidence of an effect of OTA on intracellular calcium (Ca^2+^) homeostasis [74, 80–82]. Ca^2+^ homeostasis modulation by OTA was also observed *in vivo* [83]. An increased rate of microsomal Ca^2+^ uptake was observed after OTA administration *in vivo* and *in vitro* [84]. Gene profiling analysis suggested a modulation of genes involved in calcium homeostasis by OTA [47, 85]. These data indicate that Ca^2+^ dependent signal transduction pathways may be affected by OTA treatment. Interferences were observed with other cell signaling pathways. OTA was shown to stimulate phosphorylation of ERK1/2, SAP/JNK, and p38 using *in vitro* models [82, 86, 87]. Using an *in vivo* model ERK1/2 a specific signaling response was also observed with mobilization of the atypical protein kinase C (PKC*ζ*) and the insulin-like growth factor-1 (IGF-1) system [88]. Gene profiling data supported a potential role of the (IGF)-PI3K-PKB pathway in OTA-mediated renal toxicity in male rats [89]. 

### 5.1. Redox Modulation of Transcription Factors

Numerous reports have characterized interactions of ROS and RNS with activity of transcription factors [90–92]. Transcription factors contain a conserved redox sensitive cysteine residue; the oxidation of this residue inactivates the DNA-binding domain of the factor. Several studies have observed an inactivation of transcription factors resulting from increased concentrations of ROS [93–95]. For example, it was shown that the complex AP-1 is a basic leucine zipper protein, which is highly sensitive to changes in redox environment due to a cysteine residue in the DNA-binding domain [96]. Gene expression profile studies performed using *in vivo* models have shown that OTA impairs the antioxidant defense system regulated by Nrf2 [47]. This effect was supported by further studies [44–46]. Using *in vitro* models together with electrophoretic mobility shift assay, an inhibition of Nrf2 and AP-1 activity was shown as a result of OTA treatment [46]. Interestingly, Nrf2 binding site represents a bZip domain that interacts with the Antioxidant Response Element (ARE) DNA-binding site triggering the transcription of Nrf2 regulated genes. The ARE motif (5′-A/GTGAC/TnnnGCA/G-3′) shares structural similarity with AP-1 binding site (5′-TGACTCA) involving both cysteine rich residues, the target of ROS oxidation [97, 98]. These data strongly suggest a correlation between the generation of ROS by OTA and the subsequent inactivation of Nrf2 as previously described for AP-1. This molecular mechanism appears as a likely molecular component explaining the reduction of the defense response observed under OTA treatment.

*In vivo* experiments provided direct evidence that S-nitrosylation can interfere with transcription [91, 92]. Nitric oxide (NO) induces the nitrosylation of cysteine residues (thiol groups) within or near the DNA-binding domain and/or insertion of the zinc finger, which is a DNA-binding motif, resulting in the inhibition of the DNA-binding activity of transcription factors [99–101]. This is illustrated by the example of the suppression of P450 gene expression by NO. NO-donors were found to suppress CYP2D6 promoter activity through inhibition of the transcription factor called hepatocyte nuclear factor 4 (HNF4) [94, 95]. Interestingly, in a gene expression study performed by our group, OTA was associated with a significant reduction of the expression of genes regulated by HNF4 suggesting an indirect evidence of the role oxidative stress and the transcription factors regulation by OTA [47].

### 5.2. Alterations of Gap-Junction Intercellular Communication (GJIC)

A strong correlation between the ability of a compound to block cell-to-cell communication in cultured cells and its ability to induce rodent tumors through nongenotoxic mechanisms has been documented [33, 128–131]. Disruption of gap junction intercellular communication was specifically reported in human renal cancer cell lines [132]. For example, Connexin 32 (Cx32) was discovered to be generally downregulated in human renal cell carcinoma (RCC) cell lines and in cancerous regions of the kidney [133]. ROS such as H_2_O_2_, an established tumor promoter, is known to modulate cell-to-cell communication. Likewise, certain chemicals inducing ROS were shown to inhibit intercellular communication in a variety of cells in culture systems [33]. Data on the potential effects of OTA on GJIC are inconsistent. Kidney epithelial cells treated with OTA resulted in modulation of gap junction-mediated intercellular communication, through a reduced expression of the gap-junction protein CX43 [134]. In addition, OTA strongly reduced the expression of other gap-junction proteins, CX26, CX32, and CX43, in liver of rats treated with OTA [63]. In another study, even though a strong reduction of CX43 was found in renal cells *in vitro*, OTA inhibited GJIC only in liver but not in kidney [135]. 

## 6. Prevention of OTA-Induced Oxidative Stress

### 6.1. Counteracting OTA-Mediated Reduction of Nrf2 Activity

As mentioned earlier, OTA was found to reduce the expression of antioxidant enzymes through inhibition of Nrf2 activity [46]. This reduction in antioxidant gene expression was correlated with increased oxidative damage of protein and DNA [46]. To further confirm the actual role of the reduction of defense mechanisms in the induction of cellular oxidative damage, inducers of Nrf2 were applied in cell cultures *in vitro*. All reported OTA effects at the levels of Nrf2 activity, Nrf2-regulated gene expression, and DNA damage were prevented in cell pretreated with Nrf2 inducers [46], strongly indicating a causal relationship between Nrf2 effects and oxidative damage.

### 6.2. Application of Radical Scavengers

Several studies have been performed to try to counteract the adverse effects of oxygen radicals generated under OTA-treatment. A number of molecules with various antioxidant properties were tested, including *inula crithmoides*, cyanidin 3-O-b-D-glucoside, catechins, melatonin, superoxide dismutase, catalase, and N-acetyl-L-cysteine (NAC), using *in vivo* or *in vitro* models. Protection against OTA-induced DNA damage, lipid peroxidation as well as cytotoxicity was observed [75, 79, 102, 105, 106, 116, 118, 127] further confirming the link between OTA exposure and oxidative macromolecular damage. However, up to now, the application of chemicals known to possess antioxidant properties failed to prevent the development of OTA-induced tumors in animal models. Authors reported protection against nephrotoxicity but not carcinogenicity induced by ochratoxin A, implicating two separate pathways [122, 123].

### 6.3. Application of Peroxidase Inhibitors

Indomethacin and aspirin were found to prevent OTA genotoxicity in the urinary bladder and kidney of mice [136]. These data suggested the possible co-oxidation of OTA by enzymes involved in arachidonic acid biotransformation, especially prostaglandin-H-synthase (PGHS) and/or lipoxygenase [124]. Such reactions thought to produce activated OTA metabolites are also known to generate ROS which then may induce oxidation [124].

## 7. Conclusion

The carcinogenic mycotoxin OTA has been reviewed by a number of expert groups [3–5, 14, 137]. These expert groups identified the production of oxidative stress as an important event in the mode of action of OTA-induced nephrocarcinogenicity. In the present paper, the actual evidence available supporting such hypothesis was collected and reviewed. 

It has been clearly shown that OTA generates oxidative stress predominantly in kidney through potentially direct (redox cycling) and indirect (reduction of cellular antioxidant defenses) mechanisms. Interestingly, these two mechanisms may interact with each other. The reduction of defense may amplify the impact of the direct production of radicals. Consequences resulting from the production of oxidative stress were observed at different levels. High kidney susceptibility to oxidative stress conditions may explain the target-specific toxicity of OTA. Oxidative stress has been incriminated in a number of kidney pathological pathways [138–140]. 

**Figure 1:**
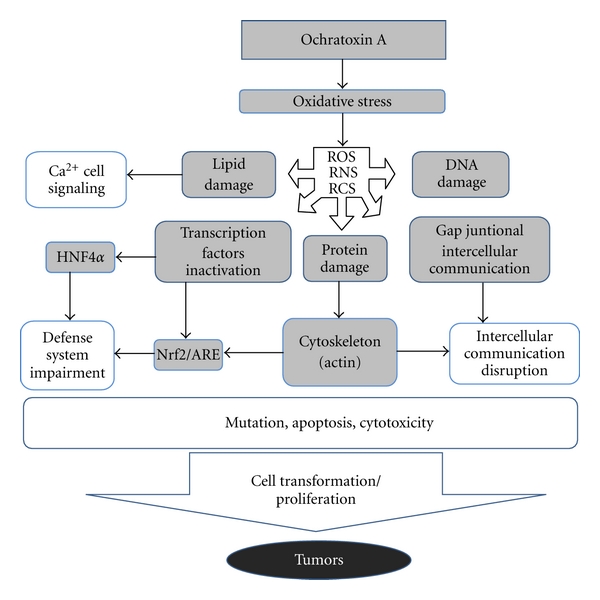
Scheme to illustrate the oxidative stress-mediated mode of action proposed for OTA. Increased production of ROS, RNS, and RCS is likely to originate either from direct redox reactions involving OTA or through the inhibition of cellular defenses such as through the inhibition of transcription factors as Nrf2 which regulates enzymes with antioxidant properties. The generation of radicals will induce macromolecular oxidative damage such as oxidized DNA bases which may be converted into mutation resulting into generation of transformed cells. In addition, radicals will trigger biological responses which may impair intercellular communication and induce cell proliferation as well as reduction in cellular defense in oxidative stress. This last effect is likely to amplify the oxidative stress-mediated effects of OTA. Altogether, these molecular mechanisms will result in cancer development.

As depicted in Figure 1, first, OTA exposure was associated with increased levels of oxidative DNA, lipid, and protein damage. Second, various biological pathways known to be mobilized under oxidative stress were shown to be altered by OTA. Importantly, these effects were observed in both *in vitro* and *in vivo* test systems. Active *in vivo* doses were within doses known to induce tumors in kidney. These mechanisms are likely to be relevant for humans. 

In conclusion, the evidence for the induction of an oxidative stress response resulting from OTA exposure can be considered strong. Because the contribution of the oxidative stress response in the development of cancers is well established, a role in OTA carcinogenicity is plausible. Altogether, the data reviewed above support the application of a threshold-based approach to establish safe level of dietary human exposure to OTA.

## Figures and Tables

**Table 1:** OTA oxidative stress-related studies.

Model	Gender	Via	Time treatment	Dose	Aim	Results/Conclusion	Ref.
BALB/c macrophage J774a cell *line *			24, 48, 72 h	30 nM–100 *μ*M	OTA immunotoxicity and modulation inflammatory process	Induction of iNOs, COX-2 and NF-*κ*b expression by OTA. OTA is an immunotoxic compound	[51]
Porcine kidney tubuli cells LL-PK1			6–24 h	1–100 *μ*mol/L	Characterization effect of OTA on Nrf2 response	Nrf2 potential signal transduction pathway by which OTA impairs its own detoxification	[45]
Porcine kidney tubuli cells LL-PK1			24 h	1–100 *μ*mol/L	Impact of OTA on Nrf2, AP-1 activity, antioxidant enzymes and GST	Enhanced production of ROS, GST impairment. Nrf2 and AP-1 disruption by OTA. Impairment of the detoxification machinery	[44]
Rat Sprague-Dawley	male	diet	15 days	3.0 mg/kg bw	Oxidative stress protection study	OTA-induced oxidative stress chemoprotection by *Inula crithmoides *	[102]
Rat F344	male	gavage	7 and 21 days	0.5 mg/kg bw	Mechanism of action study-microarrays	Oxidative stress, calcium homeostasis, cytoskeleton structure	[61]
Human hepatocytes HepG2; Monkey kidney Vero cells				0–100 *μ*M	Decrease GSH	No induction of heat shock protein (HSP)	[103]
Rat Wistar	male	diet	15 days	5 ng/g bw 50 ng/kg bw	Oxidative damage study (proteins and lipids)	Malondialdehyde (MDA) and protein carbonylation (PC) increase in kidney > liver	[76]
Chinese Hamster lung V79 cells; Lymphoma mouse LY5178 cells				0–438 *μ*M	OTA mutagenicity	OTA is mutagenic at cytotoxic doses in mammalian cells via oxidative DNA damage induction.	[104]
Rats Sprague-Dawley	male	diet	4 weeks	200 ppb	Oxidative stress protection study	OTA-induced oxidative stress and DNA damage chemoprotection by *cyanidin 3-O-*β*-D-glucoside *	[105]
Pig kidney cell line LLC-PK1			24 h	0, 10, 15, 20 *μ*M	Oxidative stress protection study	OTA-induced ROS. Scavenging by cathechins (epigallocathechin gallate (EGCG) and epicatechin gallate (ECG))	[106]
Human epithelial colorectal adenocarcinoma Caco-2 cells				100 *μ*M	Effect of OTA on barrier function impairment	Loss of microdomains associated with tight junctions maybe due to oxidative events	[107]
Neural stem/progenitor cells (NSCs)				0.01–100 *μ*g/mL	Vulnerability of brains mouse cells to OTA	Robust increased in total and mitochondrial SOD activity. OTA impaired hippocampus neurogenesis	[108]
Rats	Male/liver and kidney	Diet (drinking water)	4 weeks	289 *μ*g/kg	Oxidative stress protection study	Melatonin protection against OTA-induced oxidative damage in liver and kidney. CoQ protective in liver.	[79]
Human renal cell line HK-2			6 and 24 h	50 *μ*M	Mechanism of action study-microarrays	Significant increase in ROS level and oxidative DNA damage.	[61]
Human renal proximal tubular epithelial cell line HK-2				50–800 *μ*M	Evaluate single-strand DNA breaks and oxidative damage induction by OTA	Oxidative stress precedes cytotoxicity and genotoxicity	[57]
Male Fischer 344; Primary hepatocytes; adherent proximal tubules epithelial NRK cells; rat liver RL-34			Rats 2 years; *in vitro* culture 24–48 h;	300 *μ*g–100/kg bw; 1.5–6.0 *μ*M	Demonstration of cellular defense reduction by OTA	OTA induces depletion of antioxidant defense by inhibition of Nrf2 responsible of oxidative stress response	[46]
Eker and wild type rats	male	gavage	1–14 days	210 *μ*g/kg bw	Early carcinogen-specific gene expression study	Oxidative DNA damage response genes, general stress response, and cell proliferation	[109]
Wistar rats		gavage	90 days	289 *μ*g/kg bw	Early effects of chronic OTA administration in liver	Reduction in the ability to counteract oxidative stress in liver	[63]
Swiss ICR	male	i. p	6, 24, 72 hours	0–6 mg/kg bw	Oxidative stress and OTA neurotoxicity	Acute depletion of striate DA on a background of globally increased oxidative stress and transient inhibition of oxidative DNA repair	[110]
Swiss mice	male	I.p; infusion	2 weeks	Acute 3.5 mg/kg; chronic 4, 8, 16 mg/kg	Effect of chronic low dose OTA exposure on regional brain oxidative stress and stratial DA metabolism	Low doses exposure caused global oxidative stress	[111]
F344 rats	male	diet	7 and 21 d; 4, 7 and 12 months	300 mg/kg bw	OTA mechanism of action-microarrays study in liver and kidney	Oxidative stress and metabolic response modulated involving mainly Nrf2 and HNF4*α* pathway disruption	[47]
Swiss mice	male	oral	24 hours	10 mg/kg	Immune cells response after acute OTA exposure	OTA-induced oxidative stress response responsible of its own toxicity.	[112]
Wistar rats	female	Intraperitoneally	7, 14 and 21 days	0.5 mg/kg bw/day	Genotoxic potential of OTA measuring DNA strand breaks (comet assay) in the kidney	OTA-induced DNA strand breaks were detected, OTA concentration in the kidney and duration of the treatment correlated with severity of the DNA damage	[62]
Wistar rats	male	Oral	15 days	5 ng; 0.05 mg; 0.5 mg/bw	Effect of OTA on DNA damage	Oxidative stress responsible for OTA-DNA damage as shown by Fpg-modified comet assay	[113]
Pig kidney microsomes, human bronchial epithelial cells, human kidney cells			Cells: 2, 7, 24 hours	0.5, 1.0, 2.5 *μ*M	Genotoxicity of the hydroquinone (OTHQ) metabolite of OTA	OTQ-mediated adduct spots form in a dose-and-time-dependent manner	[114]
Wistar rats	female	oral	7, 14 and 21 days	0.5 mg/kg bw	Effect of OTA on protein oxidation in kidney and liver	Increased protein carbonyls in the kidney and liver	[68]
F344 rats	male	gavage		0.03, 0.10, 0.30 mg/kg bw	Evaluate relevance of OTA-induced oxidative damage on nephrotoxicity and carcinogenicity	Tumours in rat kidney may be attributable to oxidative DNA damage in combination with cell-specific cytotoxic and proliferation-stimulating effects as cell-signaling response	[69]
V79 (Chinese hamster lung fibroblasts) cells, CV-1 (African green monkey, kidney) cells, primary rat kidney cells			1–24 hours	2.5, 100 *μ*mol/L OTA	Relevance of OTA-induced oxidative damage in nephrotoxicity and carcinogenicity	Cytotoxicity and oxidative DNA damage already at low doses could be a relevant factor for the nephrocarcinogenicity	[58]
Rat lymphoid cells			1 hour	0.5, 2, 20 *μ*M	OTA immune function modification	Protein synthesis inhibition, oxidative metabolism of OTA, prostaglandin synthesis implicated in NK cells toxicity	[115]
Human hepatoma—derived cell lines (HepG2), human colonic adenocarcinoma cell line (Caco-2)			24, 48, 72 hours	0–100 *μ*M	Oxidative stress protection study	OTA-induced oxidative stress damage. Protective effect by Cyanidin-3-*O*-*β* glucopyranoside (C-3-G)	[116]
F344 Fischer rats	male	gavage	2 weeks	0–2000 *μ*g/kg bw	Genotoxicity of OTA	DNA strand breaks in target and nontarget tissues probably involving oxidative stress mechanism	[60]
Human hepatoma—derived cell line (HepG2)			48–72 hours	35–10 mM	Oxidative damage protection study	No cytotoxicity protection observed with Vitamine E, polyphenols	[117]
Sprague-Dawley	male	diet	15 days	3 mg/kg	Oxidative stress protection *in vivo* study	Preventive effect against OTA-induced oxidative stress and lipid peroxidation by melatonin	[75]
Human fibroblast cells			48–72 hours	0–50 *μ*M	Oxidative stress protection study	Reduction of free radical species production and DNA damage prevention by cyanin 3-*O*-*β*-D-glucoside (C3G)	[118]
Fetal rat telencephalon aggregating cells			24–48 hours, 9 days	0–20 nM	Adverse effect of OTA in brain	Brain inflammatory response induction of HO-1, iNOs, PPAR*γ*, cytoskeletal damage	[50]
Human hepatoma-derived cell line (HepG2)			24 hours	0–40 *μ*g/mL	Genotoxicity of OTA	Dose-dependent induction of DNA single strand breaks (comet assay) and micronuclei (MN)	[119]
Primary proximal tubules renal (PT) cells, proximal tubular cell line (LLC-PK_1_)			0–24 hours	0–100 *μ*M	OTA mediated oxidative stress response in proximal tubular cells, oxidative stress protection	Oxidative stress contributes to tubular toxicity. Antioxidants (*α*-tocopherol, N-acetyl-Lcysteine (NAC) treatment prevents OTA toxicity	[59]
Wistar rats	male	gavage	10, 30, 60 days	120 *μ*g/kg bw	Kidney low dose OTA response: sequence of events leading to cell death	Low dose induces oxidative stress, apoptosis in proximal, and distal tubule kidney cells	[78]
Human hepatoma—derived cell line (HepG2)			1 hour, 24 hours	0–50 *μ*g/mL	Genotoxicity of OTA	No inductions of mutations in the Ames assay, a dose-dependent induction of micronuclei in the MN assay, and DNA migration (comet assay) were detected	[120]
Proximal tubular cells (PTC), Wistar rats	male	gavage	24–72 hours (*in vivo *and *in vitro*)	5.0 *μ*M, 12.5 *μ*M *in vitro*; 1 and 10 mg/kg bw	*In vivo* and *in vitro* gene expression comparative study	*In vitro* and *in vivo* gene expression data were comparable. Response to oxidative stress-related genes hypoxia-inducible factor 1 and catalase was observed	[121]
Dark Agouti (DA), Lewis rats	male	Intragastric intubation	0.4 mg/kg bw	Life-time	Life-time study to evaluate if MESNA leads to a more effective reduction of OTA-induced tumour development or urinary tract damage	Lack of effect of mesna on OTA-induced urinary tract damage or renal tumor development	[122]
Dark Agouti (DA), Lewis rats	male	Intragastric intubation	0.4 mg/kg bw	2 years	Life-time study to evaluate the potential protective effect of 2 mercaptoethane sulfonate (MESNA) and N-acetyl cysteine (NAC )	MESNA decreased karyomegalies in kidney, but had no beneficial effect on renal tumour incidence	[123]
Fischer rats	male	gavage	4, 8, 24, 48 hours	0–2.0 mg/kg bw	Chemical and biological markers induced by OTA exposure associated with oxidative stress	Oxidative stress may contribute to mechanism of OTA renal toxicity and carcinogenicity in rats over long term exposure	[77]
Bronchial epithelial cells incubated with microsomes of seminal vesicles of pig			4 hours	10 *μ*M	Roles of cyclooxygenase and lipoxygenase in ochratoxin A genotoxicity in human epithelial lung cells	OTA is biotransformed into genotoxic metabolite via a lipoxygenase, whereas prostaglandin—H-synthetase (PGHS) decreases OTA genotoxicity	[124]
Sprague-Dawley liver microsomes, liver mitochondria and hepatocytes cells	female			2.5 mM	Free radical generation by OTA in hepatocytes, mitochondria, and microsomes using electron paramagnetic resonance (EPR)	Oxidative damage may be one of the manifestations of cellular damage in the toxicity of OTA	[125]
*Bacillus brevis*			10 min	1 mg/mL	Study free radical generation in bacteria as model system	OTA induces free radical production, enhancing permeability of the cellular membrane to Ca2^+^	[37]
Swiss mice	Male	Gastric intubation	48 hours	2 mg/kg bw	Effects of vitamins on genotoxicity of OTA	Vitamins E, A, and C also reduced OTA-DNA adduct formation in mice kidney	[126]
Wistar rat	male	Gastric intubation	Every 48 hours/3 weeks	289 *μ*g/kg body weight	Protective effect of superoxide dismutase (SOD) and catalase	SOD + catalase prevents the nephrotoxicity induced by OTA in rats	[127]
Wistar rat liver microsomes, kidney microsomes	male			6 mg/kg bw	Lipid peroxidation induction by OTA	lipid peroxidation may play a role in the observed toxicity of ochratoxin A	[73]
